# Ferroptosis-related gene SLC1A5 is a novel prognostic biomarker and correlates with immune infiltrates in stomach adenocarcinoma

**DOI:** 10.1186/s12935-022-02544-8

**Published:** 2022-03-19

**Authors:** Dandan Zhu, Sifan Wu, Yafang Li, Yu Zhang, Jierong Chen, Jianhong Ma, Lixue Cao, Zejian Lyu, Tieying Hou

**Affiliations:** 1 School of Medicine, South China University of Technology, Guangzhou, 510006 Guangdong China; 2 Guangdong Clinical Laboratory Center, Guangdong Provincial People’s Hospital; Guangdong Academy of Medical Sciences, Guangzhou, 510080 Guangdong China; 3 The Second School of Clinical Medicine, Southern Medical University, Guangzhou, 510515 Guangdong China; 4 Medical Department, Guangdong Provincial People’s Hospital; Guangdong Academy of Medical Sciences, Guangzhou, 510080 Guangdong China; 5 Department of Gastrointestinal Surgery, Guangdong Provincial People’s Hospital; Guangdong Academy of Medical Sciences, Guangzhou, 510080 Guangdong China; 6 Research Center of Medical Sciences, Guangdong Provincial People’s Hospital; Guangdong Academy of Medical Sciences, Guangzhou, 510080 Guangdong China; 7 Department of Laboratory Medicine, Guangdong Provincial People’s Hospital; Guangdong Academy of Medical Sciences, Guangzhou, 510080 Guangdong China

**Keywords:** Stomach adenocarcinoma, CeRNA, Ferroptosis, Tumor-infiltrating immune cells, Biomarker, Immune checkpoints

## Abstract

**Background:**

Stomach adenocarcinoma (STAD) is associated with high morbidity and mortality rates. Ferroptosis is an iron-dependent form of cell death, which plays an important role in the development of many cancers. Tumor-associated competing endogenous RNAs (ceRNAs) regulate tumorigenesis and development. Our study aimed to construct ceRNA networks and explore the relationship between ferroptosis-related genes in the ceRNA network and immune infiltration in STAD.

**Methods:**

Based on the interactions among long noncoding RNAs (lncRNAs), microRNAs (miRNAs), and messenger RNAs (mRNAs), a ceRNA network was constructed to illustrate the relationships among lncRNAs, miRNAs, and mRNAs. Subsequently, gene ontology (GO) and Kyoto encyclopedia of genes and genomes (KEGG) functional enrichment analyses were carried out to explore the functions and interactions of the differentially expressed (DE) mRNAs related to the ceRNA network. Differential expression and prognostic analysis of ferroptosis-related genes in the ceRNA network were performed using the R package “limma” and “survminer.” The correlation between ferroptosis-related genes and tumor-infiltrating immune cells was analyzed using Spearman correlation analysis and CIBERSORT. Quantitative real-time PCR (qRT-PCR) was used to validate the expression of ferroptosis-related genes in STAD cells lines.

**Results:**

A ceRNA network consisting of 29 DElncRNAs, 31 DEmiRNAs, and 182 DEmRNAs was constructed. These DEmRNAs were significantly enriched in pathways related to the occurrence and development of STAD. The ferroptosis-related gene SLC1A5 was upregulated in STAD (P < 0.001) and was associated with better prognosis (P = 0.049). The CIBERSORT database and Spearman correlation analysis indicated that SLC1A5 was correlated with eight types of tumor-infiltrating immune cells and immune checkpoints, including PD-L1(CD-274) and PD-1(PDCD1). The SLC1A5 mRNA was found to be highly expressed in STAD cells lines.

**Conclusions:**

Our study provides insights into the function of ceRNAs in STAD and identifies biomarkers for the development of therapies for STAD. The ferroptosis-related gene SLC1A5 in the ceRNA network was associated with both tumor-infiltrating immune cells and immune checkpoints in the tumor microenvironment, suggesting that SLC1A5 may be a novel prognostic marker and a potential target for STAD immunotherapy in the future.

**Supplementary Information:**

The online version contains supplementary material available at 10.1186/s12935-022-02544-8.

## Background

Stomach adenocarcinoma (STAD) is the fifth most common cancer type worldwide and the third leading cause of cancer-related deaths [1]. STAD is the most common histological type of malignant tumor that originates in the stomach and is a heterogeneous disease with different phenotypes and genotypes. Although the treatment of STAD has rapidly advanced due to the development of laparoscopic technology [2], because of the absence of clear early symptoms, most patients with STAD are already at an advanced stage at the time of diagnosis and are prone to distant metastasis; thus, the prognosis remains poor [3–5]. Therefore, new STAD treatments and prognostic targets are urgently needed to improve the survival rate of these patients.

The ceRNA hypothesis [6] was first proposed in 2011, and posits that lncRNAs regulate the expression of target mRNAs by adsorbing miRNAs and thereby act as ceRNAs; they competitively bind to shared miRNAs, inhibiting the degradation of mRNA and thus acting as miRNA sponges. To date, the complex oncogenesis-related ceRNA network of lncRNA–miRNA–mRNA interactions has been explored in various types of cancer, such as colorectal cancer [7], cervical cancer [8], and lung squamous cell carcinoma [9]. However, ceRNA network analysis in patients with STAD is relatively rare.

Ferroptosis was first proposed as a new form of cell death in 2012. Ferroptosis leads to cancer cell death by regulating iron-, amino acid and glutathione-, and ROS-metabolism, especially for the removal of aggressive malignancies that show resistance to conventional therapies [10]. Ferroptotic cancer cells may influence the therapeutic effect of anti-tumor immunity by releasing signals such as oxidized lipid mediators, or some iron-sagging cells may suppress the immune system and promote the growth of tumor cells [11]. However, few studies have explored the correlation between ferroptosis and immune infiltration in STAD.

Therefore, we comprehensively analyzed and identified some RNAs, including lncRNAs, miRNAs, and mRNAs. Based on these RNAs, we constructed a ceRNA network to elucidate the lncRNA–miRNA–mRNA interactions in STAD and identified biomarkers for the development of therapies for STAD. Finally, ferroptosis-related genes were screened in the ceRNA network and subjected to differential expression and prognostic analyses, to explore the relationship between them and immune infiltration in STAD.

## Methods

### Data collection and preprocessing

We used the genomics data commons data transfer tool (https://gdc.cancer.gov/access-data/gdc-data-transfer-tool.html) to download the published the cancer genome atlas (TCGA) RNA-seq data, miRNA data, and the corresponding clinical information on STADs. The screening criteria for lncRNAs and mRNAs included "Project: TCGA-STAD," "Experimental strategy: RNA-Seq," and "Workflow type: HTSeq-Counts" which included 375 STAD tissues and 32 normal gastric tissues. The screening criteria for miRNA included "Project: TCGA-STAD," "Experimental strategy: miRNA-Seq," and "Workflow type: miRNA Profiling", which included 446 STAD tissues and 45 normal gastric tissues. The clinical follow-up datasets from 409 patients with STAD were also obtained from TCGA database.

### Analysis of the DE lncRNAs, miRNAs, and mRNAs

We used the “edgeR” package [12] to screen DElncRNAs, DEmiRNAs and DEmRNAs with thresholds of false discovery rate (FDR) < 0.01 and |log 2 (fold change [FC])|> 1. Volcano plots were generated using the “ggplots.”

### Prediction of DEmRNAs targeted by DEmiRNAs

We used the miRcode (http://www.mirco de.org/) database [13] to predict the interactions between DElncRNAs and DEmiRNAs. In addition, mRNAs targeted by DEmiRNAs were retrieved from the TargetScan (http://www.targetscan.org/), miRTarBase (http://mirtarbase.mbc.nctu.edu.tw/php/index.php), and miRDB (http://www.mirdb.org/) databases [14–16]. The mRNAs that were identified by all three databases and then overlapped with the DEmRNAs were the DEmRNA candidates. The overlapping target genes were identified through Venn overlap analysis.

### Construction of the ceRNA network

Based on the DERNAs and the relationships between the identified miRNA-mRNA and miRNA-lncRNA pairs, Cytoscape (version 3.7.2) was used to construct and visualize the ceRNA network [17].

### Functional enrichment analysis

The “ClusterProfiler” software package [18] in R software was used to perform Gene Ontology (GO) functional enrichment [19] and Kyoto encyclopedia of genes and genomes (KEGG) pathway enrichment analyses [20]. P < 0.05, was used as the threshold of statistical significance in the GO and KEGG enrichment analyses. The results were visualized using the “ggplots” package of R software.

### Screening for ferroptosis-related genes in the ceRNA network and prognostic analysis

Sixty ferroptosis-related genes were queried from the reported literature [21–24] and are shown in Additional file 1. The ferroptosis-related genes were intersected with DE miRNA-targeted genes to derive the associated genes. For the selected genes, the median expression value was used as the cut-off point, and the patients with STAD were divided into high and low-expression groups, and Kaplan–Meier survival curves were drawn. The log-rank test was used to compare the difference in survival time between the high and low-expression groups. A similar analysis was performed for upstream miRNAs. Finally, we performed univariate and multivariate analyses of ferroptosis-related genes using the R package "survival" to identify their prognostic significance.

### Gene set enrichment analysis (GSEA)

In the TCGA cohort, we divided the 375 patients with STAD into two groups according to the median expression values of ferroptosis-related genes and chose the h.all.v6.2.symbols.gmt in the Molecular Signatures Database (MSigDB) as the reference gene set to perform GSEA analysis.

### CIBERSORT estimation and immune-related analysis

We used the CIBERSORT algorithm to evaluate 22 immune cell types in STAD. Samples were only used for further analysis when the CIBERSORT output p < 0.05. To show the correlation of various immune cells, a co-expressed heatmap was drawn based on the results of the Spearman correlation analysis. The Wilcoxon rank-sum test revealed statistically significant differences in the proportion of immune infiltrating cells between the two groups with high and low expression of ferroptosis-related genes (p < 0.05). Spearman correlation analysis was performed for the selected ferroptosis-related biomarker in the ceRNA network and the proportion of each related immune cell with p < 0.05. Immune cells differentially expressed in the high and low groups of ferroptosis-related genes were intersected with immune cells associated with the expression of ferroptosis-related genes using the R package “VennDiagram” to obtain immune cells associated with ferroptosis-related genes. Spearman correlation analysis was used to assess the correlation between ferroptosis -related genes and the expression of immune checkpoints PD-1, PD-L1 and CTLA4. Finally, we downloaded two immunotherapy cohorts, the IMvigor210 cohort of atezolizumab (anti-PD-L1 antibody) for advanced metastatic cell carcinoma [25] and the GSE78220 cohort of pembrolizumab (anti-PD-1 antibody) for melanoma [26]. The correlation of iron death-related gene expression with anti-PD-L1 and PD-1 treatment response was analyzed in these two immunotherapy cohorts, respectively, and P < 0.05 was considered statistically significant.

### Cell lines and cell culture

STAD cell lines AGS, MGC-803, SGC-7901, BGC-823, MKN-45, MKN-28, HGC-27, and human gastric epithelial cells (GES-1) were purchased from ATCC (American Type Culture Collection, Manassas, VA, USA). All STAD cell lines were cultured in 1640 medium (Gibco, Gaithersburg, MD, USA) supplemented with 10% fetal bovine serum (FBS, Gibco-BRL, Paisley, UK), 100 U/mL penicillin, and 100 μg/mL streptomycin at 37 °C in 5% CO_2_.

### RNA extraction and quantitative real-time PCR (qRT-PCR)

Total RNA was extracted from cell lines using TRIzol® Reagent (Invitrogen, Carlsbad, CA, USA). Total RNA was reverse transcribed into cDNA using PrimeScript™ RT Master Mix (Takara, Dalian, China) and then used to perform qRT-PCR with SYBR® qPCR Master Mix (Vazyme, Nanjing, China). Glyceraldehyde 3-phosphate dehydrogenase (GAPDH) was used as the internal control for gene quantification. The 2^−ΔCT^ value was calculated for every sample and normalized to that of GAPDH. The primer sequences used for PCR are listed in Additional file 2.

### Cell Counting Kit-8 assay

Cell Counting Kit-8 (CCK-8, Dojindo Laboratories Kumamoto, Japan) for cell proliferation analysis, according to the manufacturer's instructions. Cells were grown in each well of a 96-well plate at a density of 2 × 10^3^ cells/well. Afterwards, 100 ul of CCK-8 solution (CCK-8 solution was prepared from 10 ul of CCK-8 reagent and 100 ul of culture medium) was added to each well at different time points (24 h, 48 h, 72 h and 96 h), and the absorbance was measured at 450 nm after incubation at 37 °C for 2 h.

### Transwell assay

For cell migration assays, Transwell chambers (Corning, USA) with 0.8um pore size were placed in 24-well plates. In the lower chamber, 1640 containing 10% fetal bovine serum was added, and then MGC-803 cell line transfected with siRNA were inoculated with serum-free medium in the upper chamber at a density of 5 × 10^4^ cells/well and incubated for 48 h at 37 °C. Cells that migrated to the lower chamber were fixed with 4% paraformaldehyde for 30 min and then stained with 1% crystal violet for 30 min. The unmigrated cells at the bottom of the chamber were gently wiped with a cotton swab and the stained cells that had migrated to the lower chamber were photographed with a light microscope.

### Apoptosis detection by flow cytometry

MGC-803 cell line transfected with siRNA were collected, stained with FITC Annexin V and propidium iodide (BD, USA) and analyzed for apoptosis by flow cytometry (BD, USA). PI-negative and FITC Annexin V-positive cells identified apoptosis at an early stage, while late or already dead cells were positive for both FITC Annexin V and PI. The results were analyzed by FlowJo software.

## Results

### Identification of DEmRNAs, DElncRNAs and DEmiRNAs between STAD tissues and normal gastric tissues

**Fig. 1:**
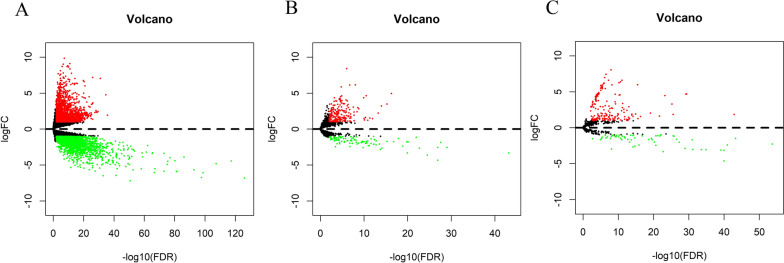
Differentially expressed RNAs (DERNAs) in stomach adenocarcinoma. Volcano plots of **A** DEmRNAs, **B** DElncRNAs and **C** DEmiRNAs. Upregulated transcripts are shown in red and downregulated transcripts in green. DE: differentially expressed; lncRNAs: long noncoding RNA; miRNAs: microRNAs; FC: fold change; FDR: false discovery rate

By applying the screening criteria, we identified 4343 DEmRNAs (2191 upregulated and 2152 downregulated) between STAD and normal gastric tissues. We identified 327 DElncRNAs (224 upregulated and 103 downregulated) and 242 DEmiRNAs (178 upregulated and 64 downregulated) in STAD tissues compared with normal gastric tissues. The corresponding volcano plots are shown in Fig. 1.

### Prediction of DEmRNAs targeted by DEmiRNAs

**Fig. 2:**
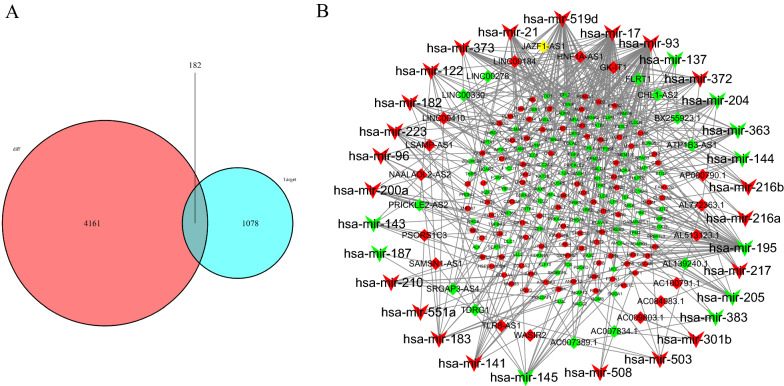
Competitive endogenous RNA (ceRNA) networks in stomach adenocarcinoma. **A** Venn diagram of mRNAs involved in the ceRNA regulation network. Red area: number of DEmRNAs; Blue area: number of mRNAs targeted by DEmiRNA; Purple area: number of mRNAs that are differentially expressed and targeted by DEmiRNA. DEmRNAs: differentially expressed mRNAs; DEmiRNA: differentially expressed microRNAs. **B** ceRNA network between DElncRNAs and DEmRNAs mediated by DEmiRNAs in stomach adenocarcinoma. The round, rhombus, and triangle nodes represent DEmRNAs, DElncRNAs, and DEmiRNAs, respectively. Red indicates high expression, whereas green indicates low expression

To identify the target mRNAs, we input the 31 DEmiRNAs into the TargetScan, miRTarBase, and miRDB databases. A total of 1260 mRNAs were identified as targets for the 31 DEmiRNAs. The 1260 candidate mRNAs predicted by these databases intersected with 4343 DEmRNA candidates, and 182 DEmRNAs were differentially expressed and shared as targets (Fig. 2A). A total of 309 pairs of interactions were identified between the 182 DEmRNAs and 31 DEmiRNAs. Only DEmiRNAs that interacted with DEmRNAs and DElncRNAs were selected to construct the ceRNA network. In summary, 31 DEmiRNAs, 182 DEmRNAs, and 29 DElncRNAs were used to construct the ceRNA network.

### Construction of the ceRNA network

A lncRNA-miRNA-mRNA ceRNA network containing 242 molecules and 440 pairs of interactions (131 pairs of DEmiRNA–DElncRNA and 309 pairs of DEmiRNA–DEmRNA interactions) was constructed using the data analyzed above. This network included 131 pairs of DEmiRNA–DElncRNA interactions and 309 pairs of DEmiRNA–DEmRNA interactions. Subsequently, Cytoscape (version3.7.2) software was used to visualize and map the entire network (Fig. 2B).

### Functional enrichment of DEmRNAs

**Fig. 3:**
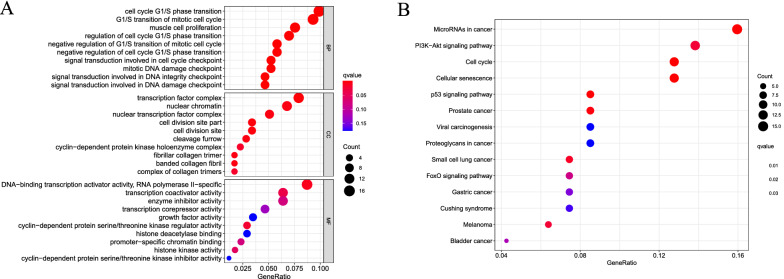
Gene ontology (GO) and Kyoto encyclopedia of genes and genomes (KEGG) analysis of differentially expressed mRNAs (DEmRNAs). **A** Bubble plot of enriched GO terms. **B** Bubble plot of KEGG

GO consists of three parts: biological processes, cellular components, and molecular functions. The top ten aspects in each part are shown in Fig. 3A. KEGG pathway analysis revealed that these DEmRNAs were enriched in a total of 14 signaling pathways (Fig. 3B). Additional file 3 represents the actual genes in the GO enriched biological processes and the KEGG pathways.

### SLC1A5 is upregulated in STAD and associated with better prognosis

**Fig. 4:**
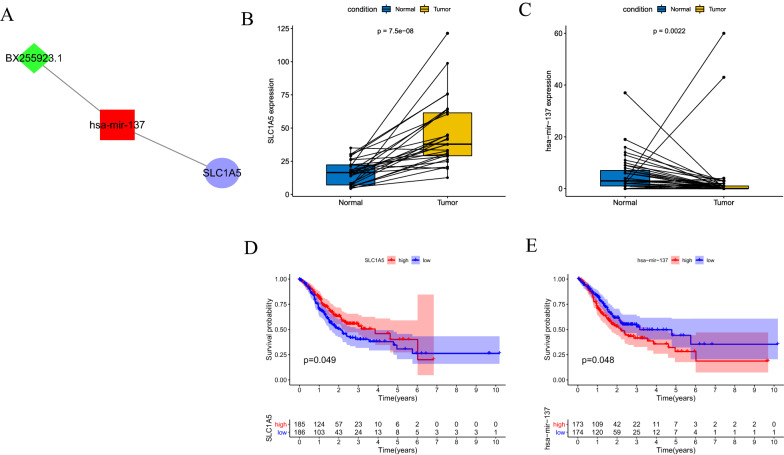
SLC1A5 and its upstream miR-137. **A** SLC1A5 and its upstream miR-137 that can be sponged by the BX255923.1. **B** The mRNA expression of SLC1A5 is significantly upregulated in stomach adenocarcinoma tissue as compared with normal gastric tissue. **C** The expression of miR-137 is significantly downregulated in stomach adenocarcinoma tissue as compared with normal gastric tissue. **D** Survival curves of TCGA data stratified by SLC1A5 mRNA expression. **E** Survival curves of TCGA data stratified by miR-137 expression

**Fig. 5:**
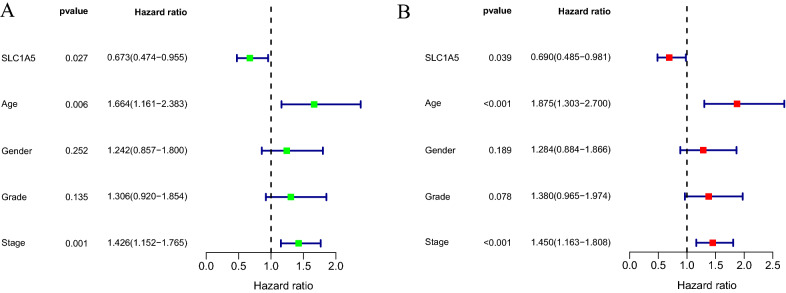
Forest plot showing univariate (**A**) and multivariate regression analysis (**B**) to determine independent prognostic factors

The 60 ferroptosis-related genes were intersected with 182 genes targeted by DEmiRNAs to obtain the gene SLC1A5, whose lncRNA-miRNA-mRNA relationship pair is shown in the Fig. 4A. In the TCGA cohort, SLC1A5 was highly expressed in the STAD tissues compared to normal gastric tissues (p = 7.5e−08) (Fig. 4B), whereas its upstream has-mir-137 was expressed at low levels in STAD tissues (p = 0.0022) (Fig. 4C). Survival analysis showed higher overall survival of patients with high SLC1A5 expression (p = 0.049) (Fig. 4D), and lower overall survival of patients with high has-mir-137 expression (p = 0.048) (Fig. 4E). In addition, univariate (Fig. 5A) and multivariate Cox regression analyses (Fig. 5B) showed that SLC1A5 expression (HR < 1) was a protective factor, while age (HR > 1) and tumor stage (HR > 1) were risk factors.

### Gene sets enriched between high and low-expression groups of SLC1A5 in GSEA analysis

**Fig. 6:**
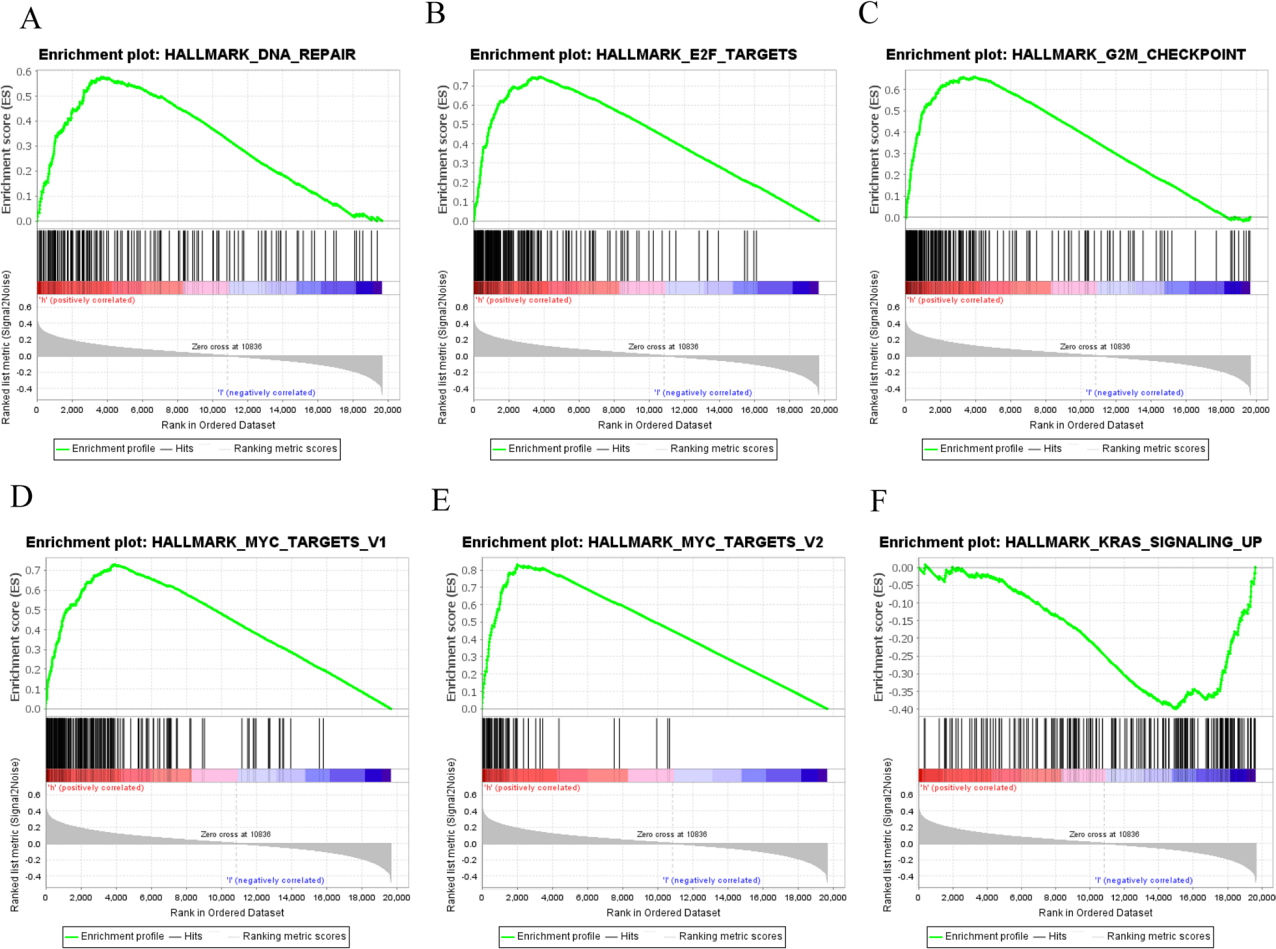
GSEA pathways using single-gene method of SLC1A5. **A**–**E** Upregulated gene sets in the high SLC1A5 expression group. **A** Enrichment plot: Hallmark_DNA_Repair. **B** Enrichment plot: Hallmark E2F_Targets_21. **C** Enrichment plot: G2M_Checkpoint. **D** Enrichment plot: Hall mark_MYC_Targets_V1. **E** Enrichment plot: Hallmark_MYC_Targets_V2. **F** Downregulated gene sets in the group of high SLC1A5 expression: Enrichment plot: Hallmark_KRAS_Signalling_Up

In the SLC1A5 high expression group, 27 (out of 50) gene sets were upregulated, 12 of which were significantly enriched with a p-value less than 0.05. In addition, three gene sets were significantly enriched in the SLC1A5 low-expression group, with p-values less than 0.05. The significantly upregulated hallmark gene sets associated with tumorigenesis in the high SLC1A5 expression group included “DNA Repair”, “E2F Targets”, “G2M Checkpoint”, “MYC Targets V1”, and “MYC Targets V2”. In the low SLC1A5 expression group, the significantly upregulated hallmark gene set was “KRAS Signaling up”, which is involved in the immune response. Snapshots of the enrichment results are shown in Fig. 6.

### Composition of tumor-infiltrating immune cells between low- and high- SLC1A5 expression groups

**Fig. 7:**
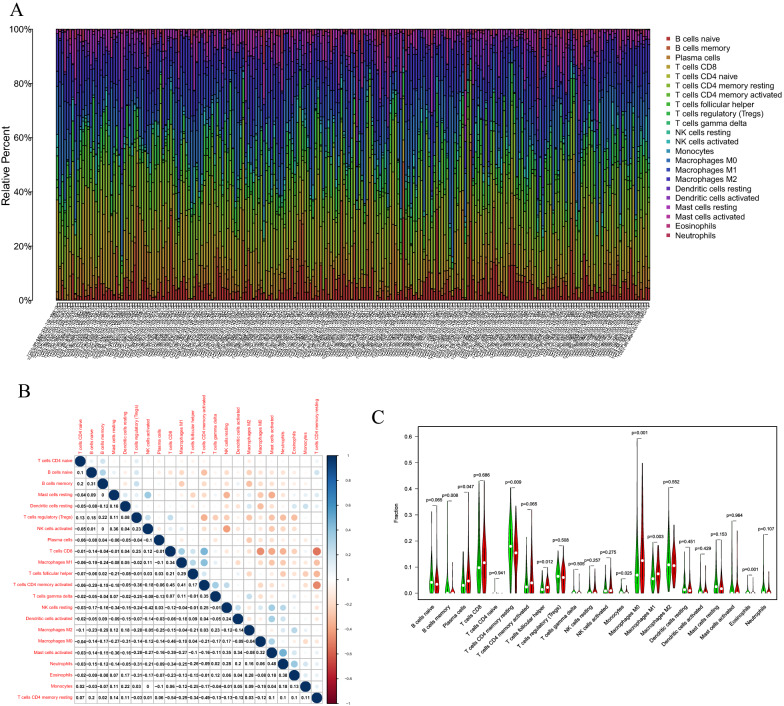
**A** The proportion of immune cell composition in each stomach adenocarcinoma sample was determined by the CIBERSORT algorithm. **B** Co-expression analysis of 22 tumor-infiltrating immune cells in STAD samples. **C** Significant tumor-infiltrating immune cells were found between the high and low SLC1A5 expression groups in STAD samples

We evaluated the composition of the significant tumor-infiltrating immune cells in STAD tissues using the CIBERSORT algorithm, and the results are shown as a histogram (Fig. 7A). Co-expression analysis using tumor-infiltrating immune cells in STAD samples was performed (Fig. 7B). Furthermore, the Wilcoxon rank-sum test indicated that B memory cells (P < 0.001), plasma cells (P = 0.047), T cells CD4 memory resting (P = 0.009), T cells follicular helper (P = 0.012), monocytes (P = 0.025), macrophages M0 (P = 0.001), Macrophages M1 (P = 0.003), and eosinophils (p = 0.001) showed significant differences in the immune cell fractions between the low- and high-SLC1A5 expression groups (Fig. 7C).

### Correlation of SLC1A5 expression with tumor-infiltrating immune cells in the tumor microenvironment

**Fig. 8:**
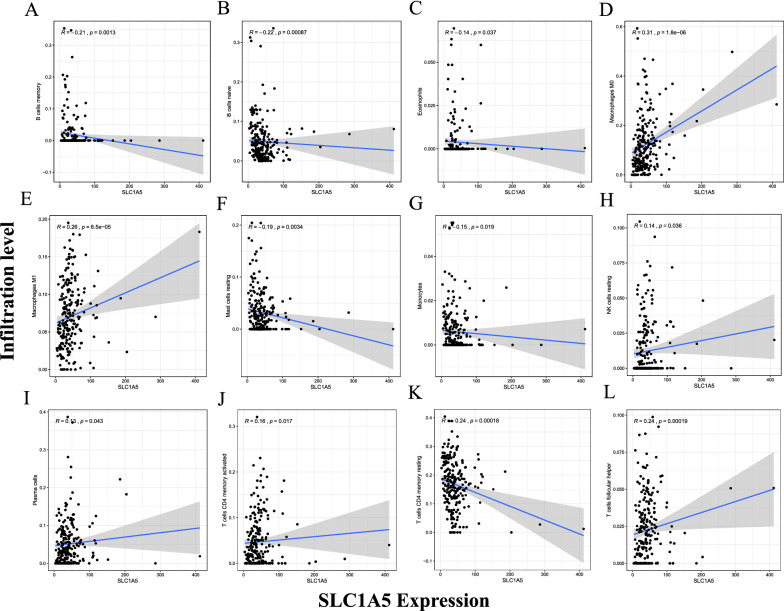
Significant correlation between SLC1A5 expression and immune cells. Significant correlation was found between SLC1A5 expression and immune cells including B memory cells (**A**), B cells naïve (**B**), eosinophils (**C**), macrophages M0 (**D**), macrophages M1 (**E**), mast cells resting (**F**), monocytes (**G**), NK cells resting (**H**), plasma cells (**I**), T cells CD4 memory activated (**J**), T cells CD4 memory resting (**K**), and T cells follicular helper (**L**)

**Fig. 9:**
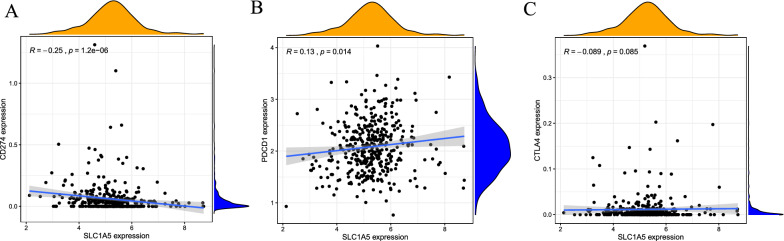
Correlation of SLC1A5 expression with PD-L1 (CD274) (**A**), PD-1 (PDCD1) (**B**), and CTLA4 expression (**C**) in the TCGA cohort

To explore the correlation between SLC1A5 expression and tumor-infiltrating immune cells, Spearman correlation analysis was performed with a p-value < 0.05. There was a significant correlation between SLC1A5 expression and the different types of immune cells, including naïve B cells (p < 0.001, cor = − 0.22), B memory cells (p = 0.001, cor = − 0.21), plasma cells (p = 0.043, cor = 0.13), T cells CD4 memory resting (p < 0.001, cor = − 0.24), T cells CD4 memory activated (p = 0.017, cor = 0.16), T cells follicular helper (p < 0.001, cor = 0.24), resting natural killer (NK) cells (p = 0.036, cor = 0.14), and eosinophils (p = 0.037, cor = − 0.14) (Fig. 8). Eight DE immune cells were intersected with 12 related immune cells to obtain eight immune cells, which were related to SLC1A5 expression. Including B memory cells, plasma cells, CD4 memory resting T cells, follicular helper T cells, monocytes, macrophages M0, macrophages M1 and eosinophils (Additional file 4). Spearman correlation analysis in TCGA-STAD cohorts of SLC1A5 with immune checkpoints showed that SLC1A5 expression was negatively correlated with PD-L1 (CD274) expression (R = − 0.25, p = 1.2e−06) (Fig. 9A) and positively correlated with PD-1 (PDCD1) expression (R = 0.13, p = 0.014) (Fig. 9B). However, the correlation between SLC1A5 and CTLA4 was not statistically significant (p = 0.085) (Fig. 9C). In the anti-PD-1 cohort (GSE78220 cohort), patients with low SLC1A5 expression were responsive to anti-PD-1 therapy (p = 0.025), but in the anti-PD-L1 cohort (IMvigor210 cohort), the expression of SLC1A5 was not statistically significantly associated with response to anti-PD-L1 therapy (p = 0.11) (Additional file 5). This suggests that SLC1A5 may be a predictive marker for anti-PD-1 therapy. In summary, the above results suggest that SLC1A5 may be involved in the immune response in the tumor microenvironment by affecting immune cell composition and immune checkpoint expression in STAD.

### Validation of SLC1A5 expression in STAD cell lines by qRT-PCR

**Fig. 10:**
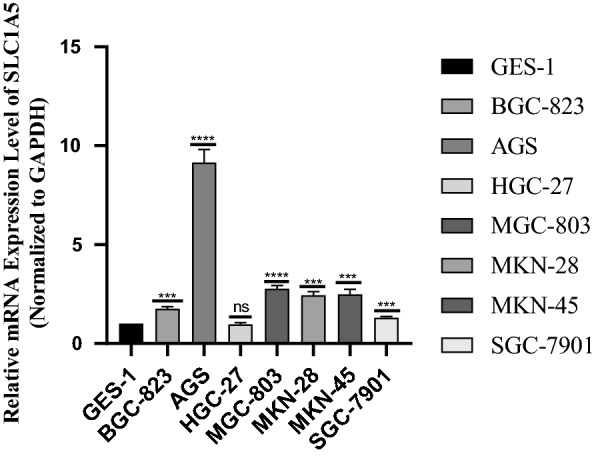
qRT-PCR validation of SLC1A5 expression in stomach adenocarcinoma (STAD) cells compared to control cells. *p < 0.05. **p < 0.01. ***p < 0.001

qRT-PCR was used to validate the expression of SLC1A5 in seven STAD cell lines (AGS, MGC-803, SGC-7901, BGC-823, MKN-45, MKN-28, and HGC-27) and one human gastric epithelial cell line (GES-1). The results showed that SLC1A5 was highly expressed in most STAD cell lines compared to that in the control cells (GES-1) (Fig. 10). This was consistent with the trend of SLC1A5 expression in TCGA cohort.

### SLC1A5 inhibits proliferation and migration and promotes apoptosis of STAD cells

**Fig. 11:**
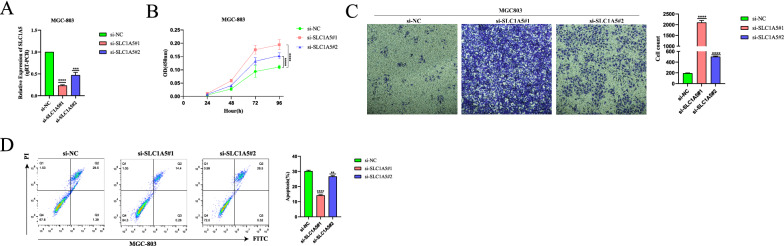
Genetic depletion of SLC1A5 promotes the proliferation and migration and inhibits the apoptosis of MGC-803 cell line. **A** Effect of knockdown of SLC1A5 expression in MGC-803 cell line after transfection with siRNA. **B** Cell proliferation assay was analyzed by the CCK-8 method each day for 4 days. **C** Transwell assay was employed to evaluate the migration effects of SLC1A5-deficient MGC-803 cell line. The cells were imaged at ×20 magnification. **D** Flow cytometry apoptosis assay showed the SLC1A5-deficient inhibits apoptosis rate in MGC-803 cell line. Data are presented as the mean ± SEM. Statistical significance was analyzed by ANOVA or Student’s t test. *p < 0.05, **p < 0.01, ***p < 0.001

We selected MGC-803, a STAD cell line commonly used to study STAD phenotypes and with high SLC1A5 expression, for Cell Counting Kit-8 assay, Transwell assay and apoptosis assay. The expression of SLC1A5 in MGC-803 cells was knocked down by transfection with siRNA (Fig. 11A). The results of the Cell Counting Kit-8 assay showed a significant increase in the proliferation capacity of the MGC-803 cell line with knockdown of SLC1A5 expression compared to the control group (Fig. 11B). Transwell assay results also demonstrated a significant increase in the number of migrated cells in the MGC-803 cell line with knockdown of SLC1A5 expression compared to the MGC-803 cell line without knockdown of SLC1A5 (Fig. 11C). We also found that knockdown of SLC1A5 expression in the MGC-803 cell line inhibited apoptosis in the flow cytometry assay (Fig. 11D). These results suggest that knockdown of SLC1A5 leads to enhanced proliferation and migration ability and reduced apoptosis ability of MGC-803 cell line.

## Discussion

The development of gastroscopy has gradually increased the rate of diagnosis of STAD, but most patients with STAD are in the progressive stage when they are detected. Thus, surgery is not effective, and the available treatment options include chemo-, targeted-, and immune-therapies. The discovery and development of immunotherapeutic agents have brought significant survival benefits to patients with STAD and are increasingly challenging the traditional treatment paradigms involving chemotherapy and targeted agents [27]. Therefore, there is a need to further understand immunotherapy-related genes as novel prognostic markers for STAD. In the present study, the ferroptosis-related gene SLC1A5 was identified as a potential prognostic biomarker for STAD, its upstream molecule miR-137 was explored, and the correlation between SLC1A5 and tumor-infiltrating immune cells and immune checkpoints in STAD was investigated. Finally, the predictive value of SLC1A5 in immunotherapy response was evaluated.

SLC1A5 is a cell surface transporter that mediates the uptake of neutral amino acids, such as glutamine [28]. The intracellular glutamine pool plays a key role in the sustained activation of the mechanistic target of rapamycin complex 1 (mTORC1) signaling, in which mTORC1 is a major regulator of cell proliferation, apoptosis, and autophagy [29]. In erastin and rsl3 induced iron death, glutamine input and metabolism induce lipid ROS production, which leads to cell death [30]. miR-137 or the inhibitor GPNA inhibits SLC1A5 and thus strongly inhibits glutamine catabolism, leading to the death of ironophilic cells. SLC1A5-mediated glutamine transport plays a crucial role in tumor cell metabolism, proliferation, and ferroptosis; therefore, inhibiting SLC1A5 and thus blocking glutamine transport is one of the approaches to treat solid tumors. miR-137 has been reported to be significantly downregulated in melanoma [31, 32], glioblastoma [33], colorectal cancer [34], and non-small cell lung cancer [35] compared to adjacent normal tissues. SLC1A5 is a target of miR-137 and is expressed at elevated levels in melanoma [36], neuroblastoma [37], and prostate cancer [38]. MiR-137 was negatively correlated with SLC1A5, suggesting that SLC1A5 is a key target of miR-137, inhibiting the growth of cancer cells. In the present study, SLC1A5 was found to be highly expressed in STAD tissues compared to normal gastric tissues, while miR-137 was expressed at low levels. In addition, the prognosis of patients with STAD was better with low miR-137 and high SLC1A5 expression, probably because low miR-137 expression attenuated the inhibitory effect on SLC1A5, thereby inhibiting the development and progression of STAD cells. This idea has not yet been suggested in any study.

Glutamine is essential for the immune system for terminally differentiated immune cells, such as neutrophils [39], macrophages [39–41], and activated lymphocytes [42, 43]. During naive T cell activation, SLC1A5 is required for rapid glutamine uptake [44], as it promotes cell growth and proliferation in T cell receptor (TCR)-stimulated mTORC1 activation [45]. SLC1A5 deletion can have an impact on T-cell effector functions, with impaired differentiation of helper T cells to Th1 and Th17 subpopulations [44]. Activated lymphocytes strongly utilize glutamine [29, 42, 46–48]. mTORC1 plays an important role in metabolic reprogramming, which is essential for NK and T cell effector functions [49–51]. And upregulation of the glutamine transporter SLC1A5 is key to mTORC1 activity [44, 52–54]. c-Myc is essential for NK cell metabolism and T cell activation [55, 56]. In T cells, c-Myc expression is required for the activation of induced glutamine hydrolysis, and glutamine uptake is critical for T cell proliferation [57]. Glutamine uptake via SLC1A5 is required for c- Myc induction in cytokine-stimulated NK cells [55]. Amino acid translocation upregulates c-Myc, while positive feedback stimulates SLC1A5 expression, maintains mTORC1 activity and supports c-Myc expression. Nevertheless, studies on the role of SLC1A5 in immune cells, which play a key role in suppressing tumor growth, are only beginning. T helper follicular cells from CD4+ T-cell subsets help B cells and induce antibody responses, thus playing an important role in anti-tumor immunity [58]. In the present study, we found that the percentage of T helper follicle cells in the high SLC1A5 expression group was higher than that in the low-expression group, and the expression of SLC1A5 was positively correlated with the content of T helper follicle cells. Monocytes are major regulators of tumor development and progression [59] and are also an important source of long-term tumor-associated macrophages (TAMs) and dendritic cells (DCs) that form the tumor microenvironment [60]. Our results showed a higher percentage of monocytes in the SLC1A5 low-expression group and a negative correlation between the two. This explains why the SLC1A5 high expression group has a better prognosis: one of the reasons may be that the infiltration of these two immune cells plays a key role.

Glutamine addiction has been reported to be one of the targets for cancer treatment by inhibiting glutaminolysis or enzymes in the glutamine transporter [61, 62]. The current research on SLC1A5 in gastric cancer treatment is also focused on glutamine metabolism. Targeting SLC1A5 in gastric cancer produces antitumor effects by inhibiting the mTOR/p-70S6K1 signaling pathway [63], glutamine mediates gastric cancer growth, and the efficacy of targeted glutamine therapy is dependent on the different expression patterns of the glutamine transporter ASCT2 and glutamate synthetase (GS) in specific gastric cancer groups [64], the new monoclonal antibody KM8094 has a very high therapeutic potential in targeting the neutral amino acid transporter ASCT2 [65, 66]. However, there are no studies that have explored the relationship between the ferroptosis-related gene SLC1A5 and immunotherapy. Immunotherapy, a therapeutic approach that boosts the immune system with drugs to fight tumors, currently plays a key role in cancer treatment [67]. Among them, immune checkpoint inhibitors (ICIs) targeting cytotoxic T-lymphocyte antigen-4 (CTLA-4) and programmed cell death protein-1 (PD-1) are promising and may play an important role in immunotherapy [68]. PD-1 is a member of the CD28 family and is essentially a suppressor receptor expressed mainly on activated T cells, B cells, macrophages, regulatory T cells (treg), and NK cells [69, 70]. It binds to two kinds of ligands, PD-L1 and PD-L2, which are mainly expressed in T cells, B cells, macrophages, and dendritic cells [71–73]. Tumors cause excessive activation of the PD-1/L1 signaling pathway, which in turn reduces T-cell activation and antigen-specific T-cell immune response, and finally bypasses immune surveillance, thus promoting tumor growth [69, 74, 75]. In the present study, we found that the expression of SLC1A5 in the TCGA-STAD cohort was positively correlated with the expression of PD-1 (PDCD1), but negatively correlated with the expression of PD-L1 (CD-274). Therefore, the better prognosis of patients with high SLC1A5 expression may be related to the reduced expression of PD-L1, resulting in fewer PD-1-binding ligands and thus a weaker immune escape effect. In contrast, immunotherapy targeting PD-1 may improve the prognosis of patients with STAD. We also confirmed the predictive value of SLC1A5 for immunotherapy response in an anti-PD-1 immunotherapy cohort. There was a significant difference in SLC1A5 expression between responders and non-responders to anti-PD-1 therapy. Although there is no published cohort of immunotherapy patients with STAD, the above results are still suggestive of SLC1A5 as a predictive marker in immunotherapy of STAD patients.

Our study is a prediction generated by preliminary data analysis and hypothesis testing, and therefore has some limitations. First, the TCGA-STAD cohort had limited number of patients and a larger sample size is required to obtain more reliable data. Second, the targeted inhibitory effect of miR-137 on SLC1A5 in STAD needs to be experimentally validated. Third, the role of SLC1A5 in regulating immune cell infiltration and immune checkpoints needs to be further investigated.

## Conclusion

In the present study, elevated SLC1A5 was found to be an independent prognostic biomarker in patients with STAD. Reduced inhibition of SLC1A5 by upstream miR-137 led to increased its expression and a better prognosis. Moreover, SLC1A5 may play an important role in the microenvironment of STAD by regulating tumor infiltration by immune cells. In addition, SLC1A5-induced high expression of PD-1 in STAD may improve the therapeutic response of patients treated with ICIs. Thus, our findings provide new insights to assist clinicians in developing appropriate therapeutic strategies and improving the long-term prognosis of STAD.

## Supplementary Information

**Additional file 1: Table S1**. Sixty genes associated with ferroptosis that have been reported in the literature.

**Additional file 2: Table S2**. Primer sequences used in the qRT-PCR assay.

**Additional file 3: Figure S1.** Gene ontology (GO) and Kyoto encyclopedia of genes and genomes (KEGG) analysis of the actual genes. (A) Circular plot of enriched GO terms. (B) Circular plot of KEGG.

**Additional file 4: Figure S2.** Venn diagram of immune cells differentially expressed between high- and low-SLC1A5 expression groups intersected with immune cells associated with SLC1A5 expression.

**Additional file 5: Figure S3.** Expression of SLC1A5 in the role of anti-PD-1/L1 immunotherapy. (A) Expression of SLC1A5 in the anti-PD-L1 clinical response group (IMvigor210 cohort). (B) Expression of SLC1A5 in the anti-PD-1 clinical response group (GSE78220 cohort).

## Data Availability

Data sharing not applicable to this article as no datasets were generated or analyzed during the current study.

## Abbreviations

STAD: Stomach adenocarcinoma
ceRNA: Competing endogenous RNA
lncRNA: Long noncoding RNA
miRNA: MicroRNA
mRNA: Messenger RNA
GO: Gene ontology
KEGG: Kyoto encyclopedia of genes and genomes
DE: Differentially expressed
qRT-PCR: Quantitative real-time PCR
TCGA: The cancer genome atlas
MSigDB: Molecular Signatures Database
ICIs: Immune checkpoint inhibitors
mTORC1: Mechanistic target of rapamycin complex 1
CTLA-4: Cytotoxic T-lymphocyte antigen-4
PD-1: Programmed cell death protein-1
