# Supplementary material for: Ferroptosis-related gene SLC1A5 is a novel prognostic biomarker and correlates with immune infiltrates in stomach adenocarcinoma
Source: Cancer Cell Int. 2022 Mar 19;22:124. doi: 10.1186/s12935-022-02544-8 (PMC8933927; doi:10.1186/s12935-022-02544-8)
Supplement: Supplementary file 2 — Additional file 2: Table S2. Primer sequences used in the qRT-PCR assay. [file 12935_2022_2544_MOESM2_ESM.pdf]

Table S2. Primer sequences used in the qRT-PCR assay

Primer	Sequence (5′–3′)
**SLC1A5-For**	ACTGCCCCTCATCTACTTCCTCTTC
**SLC1A5-Rev**	CTCCTCCACGCACTTCATCATCAG
**GAPDH-For**	CTTTGGTATCGTGGAAGGA
**GAPDH-Rev**	CACCCTGTTGCTGTAGCC
