# Supplementary figures and images for: Ferroptosis-related gene SLC1A5 is a novel prognostic biomarker and correlates with immune infiltrates in stomach adenocarcinoma
Source: Cancer Cell Int. 2022 Mar 19;22:124. doi: 10.1186/s12935-022-02544-8 (PMC8933927; doi:10.1186/s12935-022-02544-8)

A

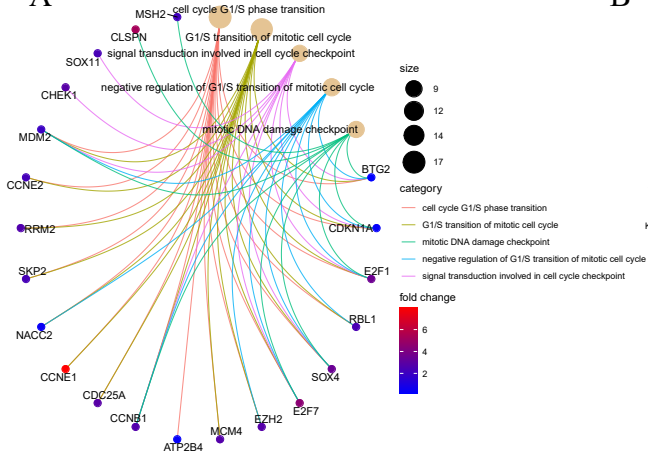

B

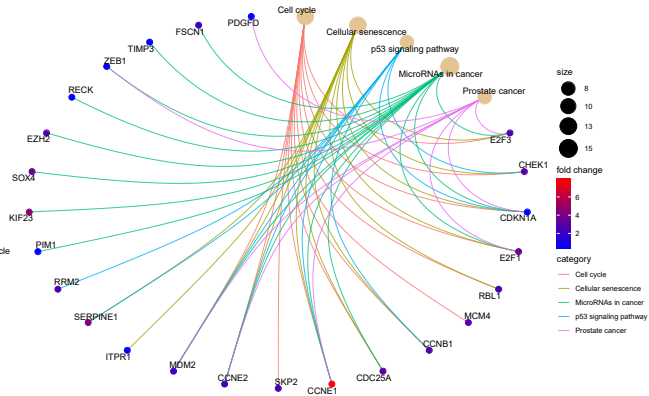

Supplement: Supplementary file 3 — Additional file 3: Figure S1. Gene ontology (GO) and Kyoto encyclopedia of genes and genomes (KEGG) analysis of the actual genes. (A) Circular plot of enriched GO terms. (B) Circular plot of KEGG. [file 12935_2022_2544_MOESM3_ESM.pdf]

# Intersection

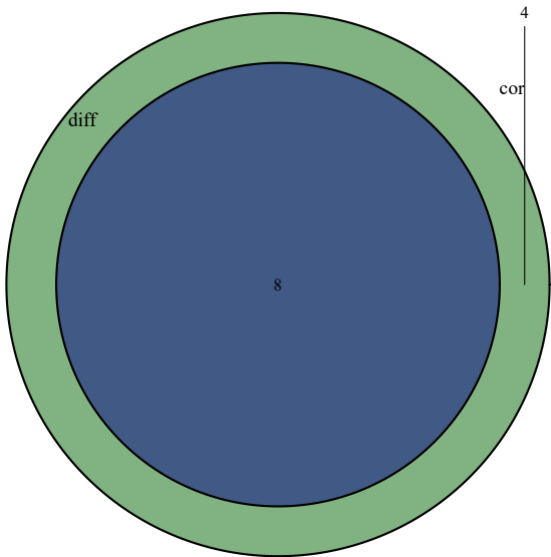

Supplement: Supplementary file 4 — Additional file 4: Figure S2. Venn diagram of immune cells differentially expressed between high- and low-SLC1A5 expression groups intersected with immune cells associated with SLC1A5 expression. [file 12935_2022_2544_MOESM4_ESM.pdf]

A

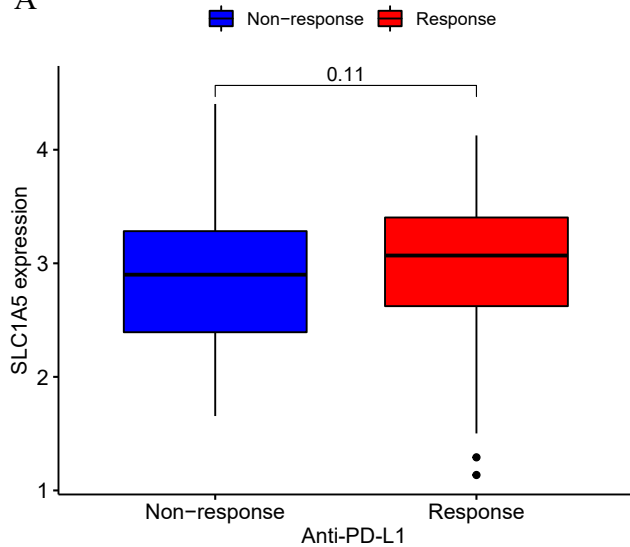

B

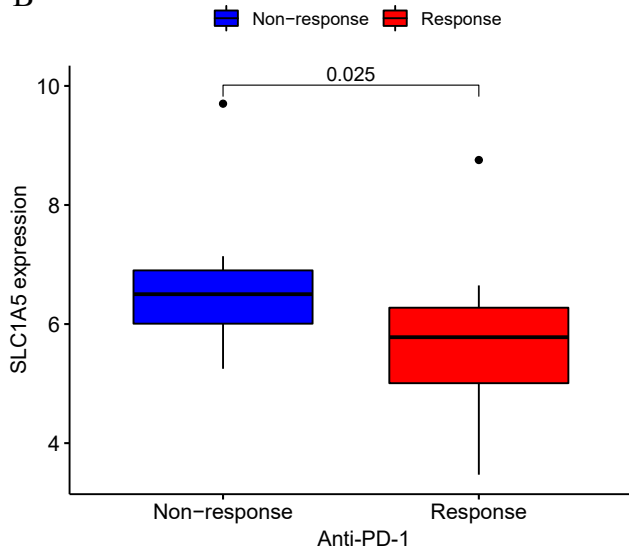

Supplement: Supplementary file 5 — Additional file 5: Figure S3. Expression of SLC1A5 in the role of anti-PD-1/L1 immunotherapy. (A) Expression of SLC1A5 in the anti-PD-L1 clinical response group (IMvigor210 cohort). (B) Expression of SLC1A5 in the anti-PD-1 clinical response group (GSE78220 cohort). [file 12935_2022_2544_MOESM5_ESM.pdf]
